# Peginterferon based therapy for chronic hepatitis C in the elderly patients

**DOI:** 10.1186/1471-2334-13-S1-P67

**Published:** 2013-12-16

**Authors:** Adriana Bold, Gabriela Iliescu, Viorel Biciuşcă

**Affiliations:** 1 University of Medicine and Pharmacy Craiova, Romania; 2 Emergency Clinical Hospital Craiova, Romania

## Background

Marrow failure and subsequent pancytopenia result from damage or suppression of pluripotent stem cells or progenitor cells, induced by drugs or viral infection. Physiologically, after the age of 65, bone marrow declines by an intrinsic reduction in cells with an increase of fat, displacing until replacement the hematologic tissue. Cytopenia may be severe and clinically significant in elderly patients with chronic C infection during alpha peginterferon based treatments.

## Methods

Ten patients, over the age of 65 (8 women and 2 men), were treated with standard peginterferon alpha 2a and ribavirin for 48 weeks. We scheduled biweekly visits during the first month and then monthly during the rest of the treatment period, as well during the initial 24-week follow-up. At each visit we performed physical examination, biochemical and hematologic tests and noted adverse events.

## Results

Only women developed severe pancytopenia - 3 patients. After the first month, hemoglobin (Hb) decreased with 1.4 g/dL. At month three, the complete blood count (CBC) showed pancytopenia with a mean platelet count of 72,000/cmm, mean Hb 9.9 g/dL, and mean leucocytes 2,000/cmm. The descending trend was constant, requiring doses adjustments and the administration of growth factors. In one patient, age 66, we discontinued the treatment. In patients with no sign of liver cirrhosis bone marrow exam was performed: it was hypocellular with a large amount of fat cells and stroma. These patients had a partial hematological response until the end of treatment: mean Hb 10.1±1.6g/dL, WBC 2,800±1500/cmm, platelets 56,000±20,000/cmm. Nine patients achieved sustained virologic response. After treatment, we decided an extended follow-up of to 5 years, marked by periods of extreme asthenia and spontaneous bleedings; there was no recovery of the cellular components of the blood and in the bone marrow.

## Conclusion

Despite frequent and severe hematological side effects, elderly patients with chronic HCV can be treated successfully with a standard-of-care regimen. Such patients require careful monitoring of blood counts and more supportive therapy.

